# P-389. Review of Post Cesarian Section Surgical Site Infections in a Tertiary Care Hospital

**DOI:** 10.1093/ofid/ofae631.590

**Published:** 2025-01-29

**Authors:** Rija B R Alvi, Clare Shanahan, Abigail Ruby, Eman Chami, Odaliz Abreu Lanfranco, Geehan Suleyman

**Affiliations:** Henry Ford Hospital, Detroit, Michigan; Henry Ford health, Detroit, Michigan; Henry Ford Health, Detroit, Michigan; Henry Ford Hospital, Detroit, Michigan; Henry Ford Health System, Wayne State University School of Medicine, Detroit, MI; Henry Ford Health, Detroit, Michigan

## Abstract

**Background:**

Surgical site infection (SSI) is one of the most common complications following cesarean section (CSEC), variably ranging from 3% to 18%, and adds a significant burden to the healthcare system. Given its substantial implications, developing strategies to prevent SSIs are essential. We aimed to explore factors associated with an increased risk of these infections.

**Figure:**
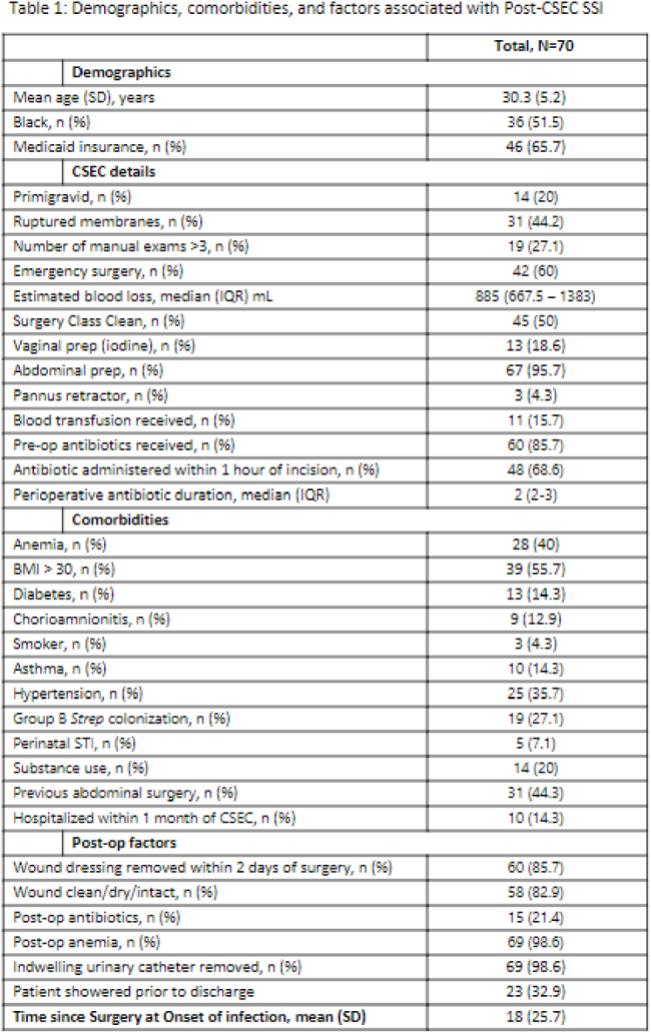

**Methods:**

Retrospective observational study of post-CSEC SSIs from Jan 2021-Dec 2023 at Henry Ford Hospital in Detroit. SSIs were defined according to the National Healthcare Safety Network (NHSN) criteria. Cases were categorized as superficial incisional (SI), deep incisional (DI) and organ space (OS). Demographics, risk factors, clinical features, treatment, and outcomes were evaluated.

**Figure:**
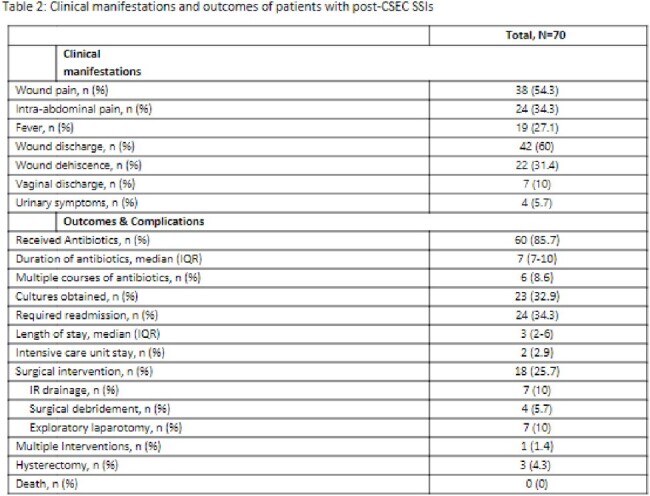

**Results:**

70 (3%) of 2,230 CSECs performed during the study period met criteria for post-CSEC SSI, of which 42 (60%) were SI, 4 (6%) DI, and 24 (34%) OS; 42 (60%) were emergency cases. The majority of patients were Black with mean age of 30.3 years (Table 1). Obesity (BMI >30), anemia (hemoglobin < 11), hypertension, prior abdominal surgery, Group B Strep colonization, and ruptured membranes at presentation were common. Most (86%) received perioperative antibiotics, and 69% were administered within 1 hour of incision; cefazolin was frequently used. Infection was diagnosed after a median of 11.5 days. Wound pain and drainage were the most prevalent symptoms. Manual chart review revealed 21% of cases did not have clinical symptoms or signs of infection. Cultures were obtained in 33% of patients, of which 91% were positive; common skin commensals were frequently isolated (Figure 1). Readmission was common, a quarter of patients required surgical intervention, and most cases were treated with antibiotics (Table 2). Complications included uterine dehiscence (9%) and half of these required hysterectomy.

Organisms Identified on cultures in patients with post-CSEC SSI

**Figure:**
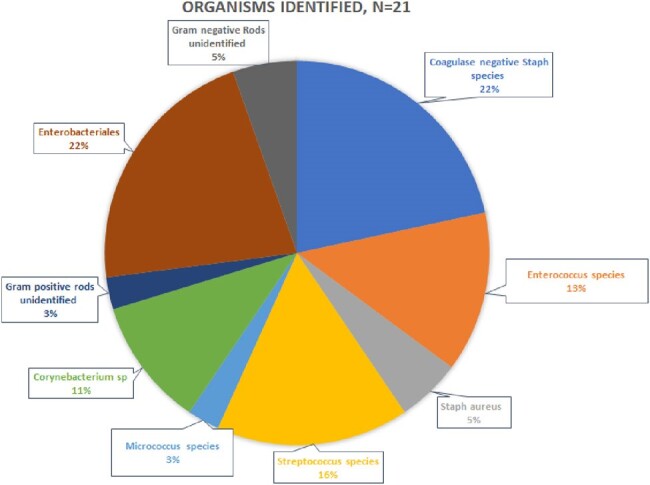

**Conclusion:**

In our cohort of patients with post-CSEC SSI, obesity and ruptured membranes were common, and more than half of the cases were performed emergently. Implementing evidence-based practices and recommendations are critical to reduce the burden of SSIs. Additionally, proper documentation and diagnostic stewardship are critical to accurately capture SSI cases and avoid unnecessary antibiotics.

**Disclosures:**

**All Authors**: No reported disclosures

